# Endoscopic third ventriculostomy for management of hydrocephalus associated with Chiari malformation type II in children

**DOI:** 10.1007/s00381-023-05832-4

**Published:** 2023-01-26

**Authors:** Nasser M. F. El-Ghandour, Mohamed M Salama, Mohamed Adel Ghoneim, Ahmed M. Attia

**Affiliations:** Department of Neurosurgery, Faculty of Medicine, Cairo University, Cairo, Egypt

**Keywords:** Lumbar puncture, Myelomeningocele, Syringomyelia, Ventriculoperitoneal shunt

## Abstract

**Background:**

Hydrocephalus is commonly associated with Chiari malformation (CM) particularly CM type II. The traditional treatment of hydrocephalus in these patients has been cerebrospinal fluid diversion by shunts. Endoscopic third ventriculostomy (ETV) has emerged as an alternative procedure in these patients.

**Purpose:**

Assessment of the clinical and radiological outcomes of ETV in the management of hydrocephalus in children with CM II.

**Methods:**

This is a prospective study conducted on 18 patients with CM II associated with hydrocephalus admitted to Cairo University hospitals between January 2020 and June 2021. These patients had been managed surgically by ETV. Clinical outcome was assessed based on improvement of manifestations of increased intracranial pressure while radiological outcome was based on the findings of postoperative computed tomography. In cases with early failure, serial lumbar puncture (LP) was performed for 2 days.

**Results:**

ETV was performed as a secondary procedure in 4 cases. The overall success rate of the procedure was 72%, and its success rate as a secondary procedure was 100%. Serial LP was effective in decreasing early failure in 44.4% of cases. Radiological regression of hydrocephalic changes was detected in 50% of the cases.

**Conclusion:**

ETV is an efficient and safe procedure in the treatment of hydrocephalus in children with Chiari malformation II, particularly when performed as a secondary procedure. Serial LP following the procedure increases the success rate in patients with early failure.

## Introduction

Chiari malformation II (CM II) refers to patients with spina bifida aperta (myelomeningocele) and hindbrain herniation. The exact incidence of hydrocephalus in CM II is not known; however, in most surgical series, the incidence of patients requiring cerebrospinal fluid (CSF) diversion reaches 80–90% [1–3]. The hydrocephalus in CM II may be due to the absence of the pulsatile flow at the level of the foramen magnum, in addition to other factors. In these cases, posterior fossa decompression may restore intracranial compliance to its normal condition and re-establish communication with the compliant spinal canal. Brain compliance may also be increased by CSF diversion due to dilatation of the compressed cortical veins resulting in decreased vascular resistance and increased cerebral blood flow [4].

Most authors would agree that coexisting hydrocephalus with CM II should be managed with CSF diversion first, either by shunting or ETV, before consideration is given to posterior fossa decompression [5, 6]. With the advent of neuro-endoscopic procedures, more and more cases of hydrocephalus are being treated by ETV to avoid shunt dependence [7, 8]. Several authors have reported the success of ETV in the management of hydrocephalus in patients with CM II with good clinical and radiological outcomes [9–12]. The success of ETV is considered when there is clinical evidence of normal intracranial pressure (ICP), avoidance of death related to hydrocephalus, avoidance of reoperation for shunt or repeat ETV within 3 months following the procedure, and structural evidence of stable or decreased ventricular size [13–15].

The aim of this study is to assess the clinical and radiological outcomes of ETV in the management of hydrocephalus in children with CM II as an alternative to the traditional CSF shunting procedures.

## Patients and methods

This prospective clinical case series was conducted on 18 children with CM II associated with hydrocephalus who were admitted to Cairo University hospitals in the period between January 2020 and June 2021. Our inclusion criteria were CM II with hydrocephalus, either de novo or with previous shunt malfunctioning, with age more than 6 months and below 18 years. We excluded patients with CSF infections, patients who had a previous ETV, and patients below 6 months of age. The data of the included patients were reviewed including patients’ demographics particularly age, clinical presentation, previous treatments for hydrocephalus, preoperative images, operative details, postoperative clinical condition, and postoperative images.

All patients had been managed surgically by ETV. We have performed the standard technique of ETV previously described by several authors [16, 17]. Patients with existing nonfunctioning ventriculo-peritoneal (VP) shunts had their shunts ligated or removed during surgery for ETV. Patients with closed heads had an external ventricular drain (EVD) inserted at the end of the procedure for repeated measuring of the ICP and as a safeguard against acute hydrocephalus and sudden death. We observed the patients for at least 48 h with monitoring of ICP in cases where an EVD was inserted. Lumbar puncture (LP) was done a maximum of 3–4 times with 12 h in between in patients with symptoms (headache, vomiting, altered conscious level) and signs (tense fontanelle, wound CSF leakage, sunken eye) of persistent increased ICP in the first postoperative days. Patients were discharged if there was an absence of pervious symptoms and signs; otherwise, a shunt was inserted in patients with persistent high pressure.

We assessed the clinical improvement of manifestations of increased tension, radiological regression of ventricular size, regression of tonsillar herniation, and improvement of the cervical syrinx. The ETV was considered successful when there was clinical evidence of normal ICP with stationary or decreased ventricular size and no reoperation for shunt or repeat ETV was needed within 3 months following the procedure. Patients were assessed in the early postoperative period, at 3 months (for detection of early failures), and at 1 year following the procedure, both clinically and radiologically. Analysis of the correlation between the patient’s age and the success of ETV as well as between previous shunt insertion (2ry ETV) and the success of the procedure was performed.

## Statistical analysis

Data was coded and entered using the statistical package for social sciences (SPSS) version 26 (IBM Corp., Armonk, NY, USA). Data was summarized using mean, standard deviation, median, minimum, and maximum in quantitative data and using frequency (count) and relative frequency (percentage) for categorical data. Comparisons between quantitative variables were done using the non-parametric Mann–Whitney test. For comparing categorical data, a chi-square (χ2) test was performed. An exact test was used instead when the expected frequency was less than 5. *P*-values less than 0.05 were considered as statistically significant.

## Results

Out of the included 18 patients, 10 patients (55.6%) were females and 8 patients (44.4%) were males with no statistical significance difference. The age of our cases ranged between 6 months and 4 years with a mean age of 11.5 months. There were 13 patients below 1 year and 5 patients above 1 year. Four patients had previous VP shunt insertion, but these shunts were nonfunctioning at the time of presentation. Patients’ demographics, clinical presentation, and associated radiological findings are shown in Table 1.

**Table 1:** Demographics and clinical presentations of the study patients

Number	**Age**	**Gender**	**Clinical Presentationr5d**	**Syrinx**
1	9 months	Male	Head enlargement, tense fontanelle	
2	6 months	Male	Tense fontanelle, leakage from meningocele wound	
3	11 months	Female	Head enlargement, vomiting	
4	8 months	Male	Sunken eye, tense fontanelle	
5	9 months	Female	Head enlargement, delayed head support	
6	6 months	Male	Tense fontanelle, vomiting, chocking	Yes
7	8 months	Male	Head enlargement, delayed head support	
8	3 years	Female	Vomiting, drowsy	
9	1 year	Male	Leakage form distal shunt wound	
10	9 months	Female	Delayed head support, tense fontanelle	Yes
11	7 months	Female	Leakage form meningocele wound	
12	4 years	Male	Tense collection around shunt reservoir	
13	9 months	Female	Head enlargement, tense fontanelle	
14	11 months	Female	Tense fontanelle, delayed head support	
15	6 months	Female	Leakage from meningocele wound	
16	1 year	Male	Head enlargement	
17	7 months	Female	Leakage from meningocele wound	
18	1 year	Female	Vomiting, delayed sitting	

Out of our 18 patients, signs and symptoms of failed ETV such as CSF leak, tense anterior fontanelle, headache, vomiting, and altered conscious level appeared in 9 patients (50%). A trial of multiple LP was performed in all of them; four of these 9 patients (44.4%) had improvement of manifestations of increased ICP. In total, 13 patients (72.2%) had improvement of their manifestations of increased ICP including those with improvement following LP, while 5 patients reported persistent symptoms suggestive of failed ETV even after LP. Follow-up of these successful cases at 3 months postoperatively revealed no recurrence of manifestations of increased ICP nor the need for further surgeries for hydrocephalus, and thus the overall success rate was 72.2%.

Radiologically, reduction of the ventricular size within a few days after surgery is a good indicator, but not a must for the success of the procedure. Regarding our cases, images of 9 patients only (50%) showed improvement of the hydrocephalic changes including one patient with no clinical improvement despite regression of ventricular size, while images of the other 9 patients were stationary. However, 55% of the patients with no radiological resolution of hydrocephalic changes had already shown clinical improvement.

We achieved success in 3 out of the 5 patients above 1 year (60%) and in 10 out of the 13 patients below 1 year (76.9%). However, the difference was not statistically significant (*p*-value = 0.07) (Table 2). Four of our cases had a history of VP shunt insertion earlier to ETV, but this shunt was not functioning at the time of our procedure. Thus, ETV was done as a secondary procedure in these cases. During surgery, the shunt was either removed (3 patients) or ligated after revision (1 patient). The ETV was successful in all of these patients (Table 3). Despite the much higher success rate of ETV as a secondary procedure, the difference between ETV as a 1ry or 2ry procedure was not statistically significant (*p*-value = 0.278).

**Table 2:** Correlation between success of ETV and age

		**Age (years)**				
		** < 1 year**		** > 1 year**		***p* value**
		**Count**	**%**	**Count**	**%**	
Clinical Improvement	Yes	10	76.9%	3	60%	0.07
	No	3	23.1%	2	40%

**Table 3:** Correlation between success of ETV and previous VP shunt

		**Previous shunt insertion**				
		**Yes**		**No**		***p* value**
		**Count**	**%**	**Count**	**%**	
Clinical Improvement	Yes	4	100%	9	64.3%	0.278
	No	0	0%	5	35.7%	

There were 16 patients who had only hydrocephalus and 2 patients who had also cervical syrinx. These two patients showed clinical improvement in the manifestations related to the syrinx as well as radiological regression of the size of the syrinx. We have also found that ETV could be effective in reducing tonsillar herniation, which was observed in 6 of our 18 patients (33.3%). Only one patient needed posterior fossa decompression due to severe lower cranial nerve affection which was still compressed by tonsillar herniation after the treatment of the hydrocephalus.

Among the 13 patients with successful ETV at 3 months, 3 patients were lost for follow-up at 1 year while 10 patients were assessed. Two of these 10 patients had reported symptoms and signs of failure after 8 months and 9 months; one of them had a VP shunt insertion, and the other had repeated ETV with choroid plexus coagulation. Thus, our success rate at 1 year was around 53% among the followed-up cases.

## Discussion

The hydrocephalus in CM II is known to be of both obstructive and communicative types. Impairment of CSF flow at the region of the fourth ventricular outlet may be the main cause of hydrocephalus in these patients. While ETV can bypass this obstruction, its role is still controversial in the treatment of hydrocephalus in Chiari patients. The question is whether ETV is an effective choice for these patients particularly those with CM II, given that previous studies had shown success rates ranging from 21 to 76% in these patients [7, 18, 19].

Our patients’ age ranged between 6 months and 4 years, with a mean age of 11.5 months. This was close to the mean age reported by Tamburrini et al. which was 16.7 months [20]. Other authors reported a wider age range and higher mean age at the time of surgery [18, 21]. On the other hand, Warf and Campbell reported mean age of 3 months [7]. We have excluded patients younger than 6 months from our study based on the high failure rates of ETV in this age group as reported by several authors. Teo and Jones have reported a 12.5% success rate in this age group as compared to an 80% success rate in older children particularly those above 2 years [18].

According to our results, there is a big difference between clinical and radiological results, so in general, we relied on the clinical assessment of manifestations of increased ICP and the status of the wound (swelling or CSF leak) for assessment of the success of ETV in follow-up. Our clinical success rate was 72% in patients with CM II at 3 months follow up but decreased to 53% at 1 year follow up. This was similar to the reported success rate of ETV in patients with CM II which ranges between 21 and 76% in several studies, with different follow up periods ranging between 2 months and 2 years. The reported success rates were higher when choroid plexus coagulation was performed in addition to the ETV [7, 18, 20–24].

In our study, the success rate of ETV was 76.9% in children below 1 year and 60% in children above 1 year. We used 1 year as a cut-off point because usually, the anterior fontanelle closes around the age of 1 year, and it is obvious that the success of ETV increases when the head is closed. According to Rei et al., the success rate below 1 year was 43% and above 1 year was 50% [21]. On the other hand, Warf has reported much higher success rates in children above 1 year, where his success rate of ETV was 47% below 1 year and 80% above 1 year [25]. Our higher success rates in children below 1 year might be explained by our exclusion of children below 6 months, whereas this category of children was included in the other studies.

The failure of ETV might occur early or as late as many years following the procedure. Our initial follow-up period was relatively short allowing for the detection of early failures only which occurred in 27.7% of our patients. Upon extension of the follow-up period to 1 year, failure was detected in around 47% of the followed-up patients. Most of our failures (71.4%) were detected in the first 3 postoperative months. Teo and Jones reported that 73% of their failures were detected within 6 weeks after surgery (early failure) [18]. Similarly, Warf and Campbell found that 86% of their failures have occurred within 6 months postoperative [7].

Several articles have addressed the role of ETV as a secondary procedure in patients with shunt malfunction irrespective of the etiology of hydrocephalus [18, 20, 24, 26, 27]. Dealing with the previous malfunctioning shunt during the ETV has been debated with no consensus about the ideal management of the already existing shunt. Teo and Jones have mentioned that leaving an open EVD following ETV may lead to a reduced pressure gradient through the ventriculostomy, thus impeding its function [28]. This might as well apply to leaving a partially functioning shunt tube un-ligated/unremoved during ETV. In their series of 20 patients with hydrocephalus of different etiologies managed by ETV following shunt malfunction, Neils et al. reported that the ETV success rate was 88% in patients whose shunts were ligated at the time of ETV, 60% in patients whose shunts were left untouched during ETV, and only 50% in patients were an EVD was used in the perioperative period [27]. We have adopted this rationale in our series where we either removed the shunt or revised it and kept it ligated to allow for the development of a pressure gradient. Similarly, in cases where an EVD was inserted at the end of the procedure, it was kept closed except when the ICP was measured.

Our study included 4 patients with previously inserted shunts who presented with manifestations of shunt malfunctioning. All these 4 patients had successful ETV (100%). A similar success rate of 100% was reported by Neils et al. in their series including 7 patients with spina bifida with shunt malfunction [27]. Teo and Jones reported that the success of ETV as a secondary procedure following shunt malfunction was significantly higher than its success as a primary procedure in patients with CM II (83% versus 29%, respectively) [18]. Tamburrini et al. have also reported higher success rates of ETV performed as a secondary procedure at the time of shunt malfunction (64.3%) in comparison to the success rate of the procedure when performed as a primary procedure (53.3%). However, it is important to point out that the mean age in the group of secondary ETV was much higher than in the group of primary ETV, at 31.8 months and 5.4 months, respectively [20]. Rei et al. reported the success of ETV in 3 out of 5 patients (60%) following shunt failure [21].

The persistent elevation of ICP observed in a group of patients in the early postoperative days is probably related to the defective absorption of the increased CSF amounts flowing out of the ventriculostomy by the subarachnoid spaces. Duru et al. performed ETV following myelomeningocele repair in 7 patients with a low success rate (14%), thus they hypothesized that the lack of fluid content, space, and pressure in the subarachnoid spaces while the defect is still open could transform into a hyper pressure zone when the spinal defect is closed tightly. This could impair the CSF flow through the stoma and enhance the spontaneous closure of the orifice, which in turn could explain a higher success rate in late ETV after the restoration of the CSF circulation [29]. Lumbar puncture has an important role in normalizing the ICP by increasing the compliance and the buffering capacities of the subarachnoid spaces, thus decreasing the CSF outflow resistance between the ventricular system and the subarachnoid spaces, resulting in decreasing of the ventricular volume and allowing faster permeation of the intracranial subarachnoid spaces. A cycle of one to three LP has been recommended in symptomatic patients with persistent ventricular dilatation after ETV, before assuming that the ETV has failed and a shunt is required [30, 31]. We have tried LP in 9 patients with persistent elevation of ICP following ETV. In 4 patients (44.4%), LP succeeded to lower the ICP to normal levels, while in the other 5 patients, the ICP returned to high levels again following the three consecutive LP necessitating the insertion of a shunt. Hu et al. have routinely performed LP for their patients following ETV at days 1 and 3. Thirty-nine patients with persistent elevation of ICP had repeated LP every other day until day 11, with normalization of the elevated ICP achieved in 35 out of these 39 patients (89.7%) [31]. Marton et al. agreed that the need for CSF removal following ETV should be reduced gradually and that the optimal reduction should be done within 3 days. However, contrary to Hu et al. practice, they reported that if there were still a need for CSF drainage after 3–4 days, the ETV would probably never work [26].

The successful ETV not only manages to lower the ICP but also to reduce syringomyelia. Our study included 2 patients with cervical syrinx; following ETV, both patients reported improvement in their symptoms related to the syrinx, and their images also showed regression in the syrinx size without the need for posterior fossa decompression or direct surgery for the syrinx itself. Similar findings were reported by other authors [32, 33]. Only one patient in our study needed posterior fossa decompression 2 months after ETV to relieve symptoms of lower cranial nerve compression by herniated tonsils. Hayhurst et al. reported posterior fossa decompression in 6 out of their 16 patients (37.5%) after a mean duration of 6 months following ETV (range: 2 weeks–12 months), while Wu et al. reported only one patient who needed posterior fossa decompression 7 years after ETV [32, 34].

In our study, the radiological improvement did not necessarily follow successful ETV, because out of the 13 patients who showed clinical improvement after ETV, images showed regression of hydrocephalic changes in only 8 patients (61.5%), while the images remained stationary in the other 5 patients (38.5%). It has been reported that the ventricular size does not necessarily improve post-operatively or indicate the success of ETV to lower the ICP. Several authors have found that almost half of their patients (53%) with clinical improvement did not show a reduction in the ventricular size which is close to our findings [35, 36]. Tamburrini et al. reported that 33.3% of their patients showed stable ventricular size but with documented CSF flow through the ventriculostomy, while Teo and Jones found that 3 patients (6%) had no change in ventricular size despite the resolution of the manifestations of increased ICP [18, 20]. We had a single patient who did not show clinical improvement although his postoperative images showed a reduction in ventricular size. Bargalló et al. have reported a similar case as one of their patients showed a reduction of the ventricular size despite no clinical improvement [36].

## Conclusion

ETV in the treatment of hydrocephalus associated with CM II is a valuable and safe procedure that helps to avoid shunt dependency with its long-term complications, particularly in children older than 6 months. The success rate of ETV in CM-associated hydrocephalus was high, particularly when ETV was performed as a secondary procedure in patients with malfunctioning shunt. It was successful not only in treating hydrocephalus, but also in the management of associated syrinx and tonsillar descent. Serial LP in the early postoperative period increases the success rate of ETV in patients with early failure, thus minimizing the need for CSF shunting procedures.

## Data Availability

The datasets generated during and/or analyzed during the current study are available from the corresponding author upon reasonable request.

## Abbreviations

CM: Chiari malformation
CSF: Cerebrospinal fluid
ETV: Endoscopic third ventriculostomy
EVD: External ventricular drain
ICP: Intracranial pressure
LP: Lumbar puncture
SPSS: Statistical packages for social sciences
VP: Ventriculo-peritoneal
